# The impact of LncRNA dysregulation on clinicopathology and survival of pancreatic cancer: a systematic review and meta-analysis (PRISMA compliant)

**DOI:** 10.1186/s12935-021-02125-1

**Published:** 2021-08-23

**Authors:** Elahe Seyed Hosseini, Ali Nikkhah, Amir Sotudeh, Marziyeh Alizadeh Zarei, Fatemeh Izadpanah, Hossein Nikzad, Hamed Haddad Kashani

**Affiliations:** 1 Gametogenesis Research Center, Kashan University of Medical Science, Kashan, Iran; 2 Anatomical Sciences Research Center, Institute for Basic Sciences, Kashan University of Medical Sciences, Kashan, Iran; 3 Student Research Committee, Kashan University of Medical Science, Kashan, Iran; 4 Food and Drug Laboratory Research Center and Food and Drug Reference Control Laboratories Center, Food & Drug Administration of Iran, MOH & ME, Tehran, Iran

**Keywords:** Pancreatic cancer, LncRNAs, Overall survival, Disease-free survival, Progression-free survival, Clinicopathological features

## Abstract

**Purpose:**

An increasing number of studies have reported a significant association between long non-coding RNAs (lncRNAs) dysregulation and pancreatic cancers. In the present study, we aimed to gather articles to evaluate the prognostic value of long non coding RNA in pancreatic cancer.

**Experimental design:**

We systematically searched all eligible articles from databases of PubMed, Web of Science, and Scopus to meta-analysis of published articles and screen association of multiple lncRNAs expression with clinicopathology and/or survival of pancreatic cancer. The pooled hazard ratios (HRs) and their 95% confidence intervals (95% CIs) were used to analysis of overall survival, disease-free survival and progression-free survival were measured with a fixed or random effects model.

**Results:**

A total of 39 articles were included in the present meta-analysis. Our results showed that dysregulation of lncRNAs were linked to overall survival (39 studies, 4736 patients HR = 0.41, 95% CI 0.25 ± 0.58, random-effects in pancreatic cancer. Moreover, altered lncRNAs were also contributed to progression-free survival (8 studies, 1180 patients HR: 1.88, 95% CI (1.35–2.62) and disease-free survival (2 studies, 285 patients, HR: 6.07, 95% CI 1.28–28.78). In addition, our findings revealed the association between dysregulated RNAs and clinicopathological features in this type of cancer.

**Conclusions:**

In conclusion, dysregulated lncRNAs could be served as promising biomarkers for diagnosis and prognosis of pancreatic cancer.

## Introduction

Pancreatic cancer (PC) is a worldwide challenging cancer characterized by poor prognosis, ranking as one of the most lethal human malignancy. The 5‐year overall survival (OS) of PC patients is less than 5%, with median survival time between 3–6 months. However, progresses in early detection, surgical techniques and treatments strategies including chemotherapy and targeted therapy have resulted in better improvements in management of PC patients, dismal prognosis of the disease has not improved over years [1].

So, it is an urgent need to identify novel diagnostic and prognostic biomarkers associated with pancreatic cancer. In recently decades Long noncoding RNAs (lncRNAs) as type of RNA that do not encode proteins with a length of > 200 nt and crucial role in several different biological processes in diverse human diseases such as development and progression of various cancers [2, 3]. Also LncRNAs play a critical physiological role in apoptosis, metastasis, invasion, migration and cell proliferation in different cancers [4, 5]. The dysregulation of different lncRNAs is reported to be potential prognostic indicators in multiple human cancers [6–9].

Previous meta-analysis has showed that high lncRNAs expression could be used as potential prognostic markers among Asian bladder cancer patients [10]. Also dysregulation of lncRNAs expression were significantly associated with clinicopathology and survival of breast cancer patients [11]. Also similar results have been reported in ovarian, cervical and prostate cancer [12–14]. In pancreatic cancer LncRNAs are identified in body fluids and are extensively found in the blood, saliva, urine, even pancreatic fluid and exosomes from tumors. And were done analysis about effect of potential of lncRNAs in the diagnosis and treatment of PC but it is not comprehensive and complete. Due to absence comprehensive article that summarize and conclude information in this field, in this study we systematically update analysis of related articles to confirm the potential prognostic value of lncRNAs in patients with pancreatic cancer. Furthermore, the association between lncRNAs and clinicopathological characteristics from published articles was investigated to update analysis rather than 2017 [15].

## Materials and methods

This systematic review and meta-analysis was done based on the standard guidelines of Preferred Reporting Items for Systematic Reviews and Meta-analysis (PRISMA) (S1 Checklist) [16, 17].

### Search strategy

A comprehensive literature search was performed by three independent reviewers (AS, AN, and ESH) through the PubMed, Web of Science, and Scopus for relevant papers published up to November 2020. The following search terms were used: ((“Long noncoding RNA”[tiab] OR “lncRNA”[tiab] OR “lncRNAs”[tiab] OR “lincRNA”[tiab] OR "lincRNAs"[tiab] OR “long non-coding RNA”[tiab] OR “long non protein coding RNA”[tiab] OR “RNA, Long Noncoding”[tiab] OR “Long intergenic non-coding RNA”[tiab]) AND (“Pancreatic tumor”[tiab] OR “Pancreatic cancer”[tiab] OR “Pancreatic neoplasm”[tiab] OR “Pancreatic carcinoma”[tiab] OR “Pancreatic malignancy”[tiab]) AND (“Prognosis”[tiab] OR “Prognostic”[tiab] OR “Predict”[tiab] OR “Survival”[tiab] OR “Overall survival”[tiab] OR “Survival rate”[tiab] OR “Outcome”[tiab] OR “Recurrence”[tiab])). Moreover, relevant articles were also reviewed manually in order to identify potentially eligible literature. No restrictions by the publication date or language were done.

### Inclusion and exclusion criteria

Articles with the following criteria were included in the current meta-analysis: (1) original study conducted on human beings, (2) literature measured the relationship between expression level of lncRNAs with clinicopathological symptom and survival rate in patients with pancreatic cancer, (3) studies which reported sufficient data to estimate hazard ratios (HRs) and their 95% confidence interval (95% CI), and (4) literature published in English. Studies were excluded if they had the following criteria: (1) insufficient data for HR and 95% CI estimation, (2) reviews, letters, laboratory articles and animal studies, (3) reported HRs for a combination of multiple lncRNAs. In addition, if a study had reported final results in different models, we included only the full-adjusted one.

### Data extraction

Data extraction was done independently by three investigators to rule out any discrepancy. The following data were extracted from each study: (1) basic information including first author’s name, year of publication and country of origin, (2) patients’ characteristic information: ethnicity, study population, sample size and follow-up duration (3) lncRNA information: names of lncRNAs, expression status, detection methods, survival results, and cut-off definition, and analysis method for survival (4) relationship between expression level of lncRNAs and survival outcome or clinicopathological characteristics (5) HRs and their 95% CIs for survival analysis. Any disagreements were resolved by discussion and consensus. The study quality was assessed via the Newcastle–Ottawa Scale (NOS) [18]. The NOS uses a star system ranging from 0 to 8 stars. Studies that achieved 7 or more stars were considered as high quality papers

### Statistical analysis

HRs and 95% CIs were obtained from studies or calculated from Kaplan–Meier survival curves using Engauge Digitizer version 4.1 [19] to calculated the overall pooled HR and 95% CI for the association between lncRNAs and survival in PC. The pooled HR was calculated using fixed-effect model, or random-effect model in cases of high between-study heterogeneity. Heterogeneity was assessed by the Cochrane Q-test, using I^2^ statistic. Heterogeneity was considered significant as P < 0.1 or I^2^ ≥ 50%. Due to high heterogeneity in results, we also were done subgroup analysis based on molecular mechanisms, ethnicity and the expression level of lncRNAs in PC. The funnel plot asymmetry test as well as the Eggers’ regression test were used to assess publication bias. Stata version 13.0 (StataCorp LP, College Station, TX, USA) was applied for the whole meta-analysis.

## Results

**Fig. 1:**
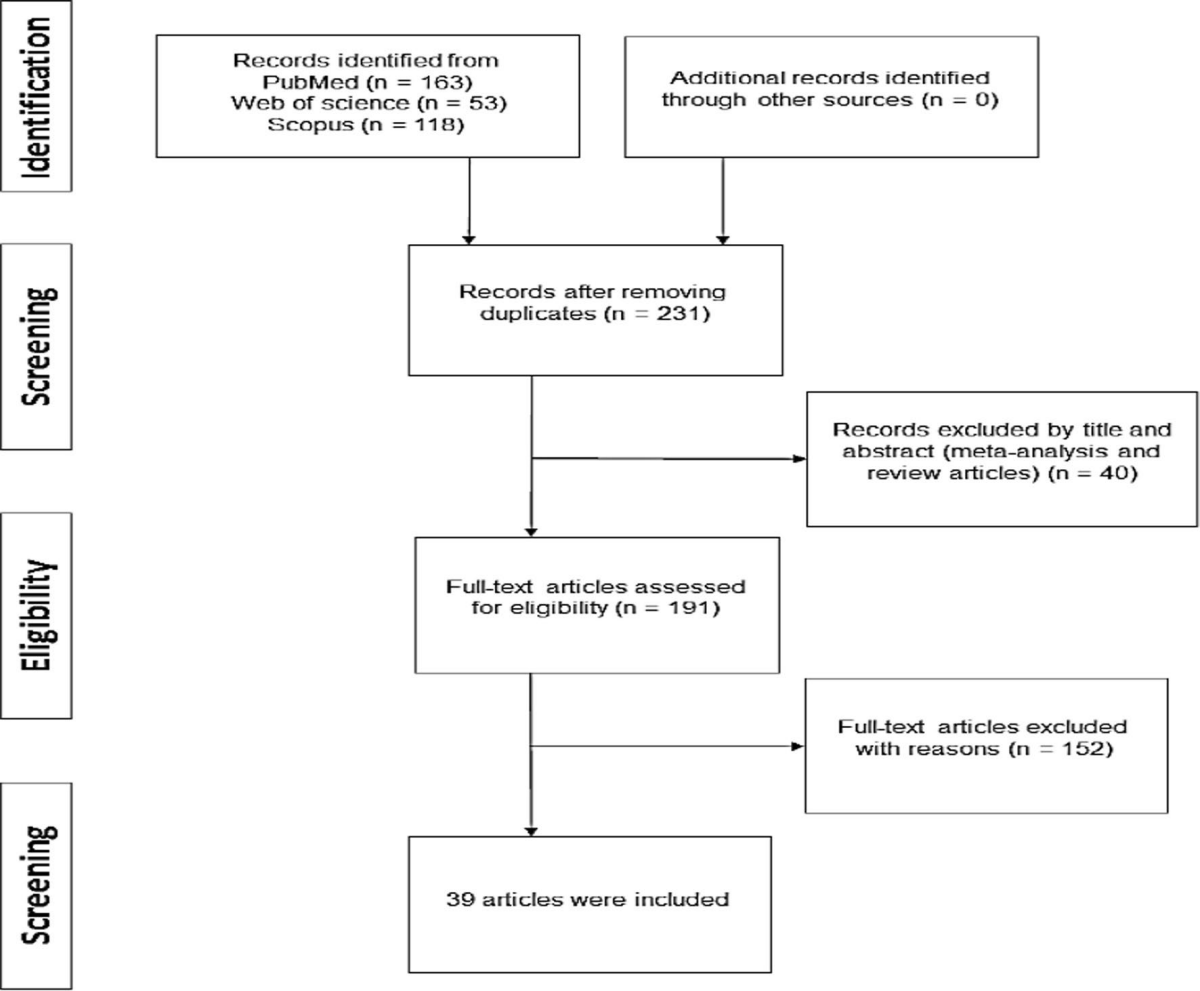
Study enrollment procedure in terms of the standards of the PRISMA diagram

As shown in Fig. 1, 336 articles were found in initial searches from PubMed, Web of Science and Scopus databases. After removing duplicate articles and screening by the title and abstracts, 191 full-text articles remained for further review. Of these, 152 studies were excluded due to insufficient data. Finally, a total of 39 studies which met our eligibility criteria were included in the current meta-analysis.

### Study characteristics

A total of 39 eligible studies involving 4736 patients diagnosed with pancreatic cancer were included in this meta-analysis. These studies were published between 2014 and 2020. Of these, 38 studies were conducted in China [20–54], and only one study in Turkey [55]. Samples were collected from tumor tissues in most studies, except three studies extracted them from blood [49], plasma [26] and serum [56]. All of these articles showed association of dysregulation of lncRNAs expression with different survival outcomes in PC. Overall survival (OS) [22–32, 34–37, 39–49, 52–59], progression free survival (PFS) [39, 56], disease specific survival (DSS) [33], and disease free survival (DFS) [36] were investigated to evaluate survival outcomes. Expression of the lncRNAs was measured by use of quantitative real-time polymerase chain reaction (qRT-PCR) using GAPDH [21, 24, 26–29, 32–35, 37, 39, 40, 42, 43, 48, 50–53, 55, 57], β-actin [23, 29–31, 45–47], RNU6B [25, 38, 41, 44], U6 [20, 22, 56] and U7 as reference genes for endogenous normalization [51].

We assessed quality of included studies using the NOS tool. Given values ranged from 4 to 8 stars based of the number of parameter that analyzed in articles: 3 study was poor quality score and awarded 4 stars [40, 57, 58], 5 study achieved 6 stars and medium quality [25, 35, 45, 46, 48], 13 studies gained 7 stars [22, 24, 30, 32, 36–38, 44, 50, 55, 59–61] and 18 articles awarded 8 stars with high quality [21, 23, 26–29, 31, 33, 34, 36, 39, 41, 43, 49, 51–53, 56, 62]. The characteristics of the included studies are summarized in Table 1.

**Table 1:** Main features of all included studies for diagnosis and prognosis

Id	lncRNAs	Author	Year	Country	Duration of research	Number of Patients (high/low)	Sum of Patients	Expression in Tumor	Internal reference	Method	Cut-off	Quality Score	Follow-up (Month)	Sample	Outcome
1	TMED11P	Zhenfei Zhu	2016	China	2007–2013	21/57	78	Down-regulation	U6	qRT-PCR	Median	7	60	Tissue	OS
2	LOC389641	Shangyou Zheng	2015	China	2008–2015	53/53	106	Up-regulation	GAPDH	qRT-PCR	Median	8	72	Tissue	OS
6	HMlincRNA717	X.L.SUN	2016	China	2005–2011	74/76	150	Down-regulation	U6	qRT-PCR	Median	7	70	Tissue	OS
7	ATB	Shibin Qu	2015	China	No	75/75	150	Down-regulation	β-Actin	qRT-PCR	Median	8	60	Tissue	OS
8	CCDC26	Wei Peng	2016	China	2011–2015	20/20	40	Up-regulation	GAPDH	qRT-PCR	Median	7	60	Tissue	OS
9	uc.345	Chao Liu	2016	China	2011–2013	68/35	103	Up-regulation	RNU6B	qRT-PCR	Median	6	50	Tissue	OS
11	Linc-pint	Le Li	2016	China	2008–2011	28/23	51	Down-regulation	GAPDH	qRT-PCR	Median	8	60	Plasma	OS
17	AFAP1-AS1	Xue-Liang Fu	2016	China	2012–2014	40/40	80	Up-regulation	GAPDH	qRT-PCR	Median	8	50–60	Tissue	OS
17	UCA1	Xue-Liang Fu	2016	China	2012–2014	40/40	80	Up-regulation	GAPDH	qRT-PCR	Median	8	50–60	Tissue	OS
17	ENSG00000218510	Xue-Liang Fu	2016	China	2012–2014	40/40	80	Down-regulation	GAPDH	qRT-PCR	Median	8	50–60	Tissue	OS
17	CRNDE	Xue-Liang Fu	2016	China	2012–2014	40/40	80	Up-regulation	GAPDH	qRT-PCR	Median	8	50–60	Tissue	OS
17	NR_036488	Xue-Liang Fu	2016	China	2012–2014	40/40	80	Up-regulation	GAPDH	qRT-PCR	Median	8	50–60	Tissue	OS
17	ENSG00000244649	Xue-Liang Fu	2016	China	2012–2014	40/40	80	Up-regulation	GAPDH	qRT-PCR	Median	8	50–60	Tissue	OS
18	UCA1	Ping Chen	2016	China	2006–2009	64/64	128	Up-regulation	GAPDH	qRT-PCR	Mean	8	60	Tissue	OS
23	HOTTIP-005	Yingxue Wang	2015	China	2006–2014	118/26	144	Up-regulation	GAPDH & β-Actin	qRT-PCR	Median	8	60	Tissue	OS
23	XLOC_006390	Yingxue Wang	2015	China	2006–2014	110/34	144	Up-regulation	GAPDH & β-Actin	qRT-PCR	Median	8	60	Tissue	OS
23	RP11-567G11.1	Yingxue Wang	2015	China	2006–2014	96/48	144	Up-regulation	GAPDH & β-Actin	qRT-PCR	Median	8	60	Tissue	OS
24	MALAT1	Er-Jun Pang	2014	China	No	63/63	126	Up-regulation	β-Actin	qRT-PCR	Median	7	60	Tissue	OS
38	ENST00000480739	Y-W Sun	2014	China	No	20/15	35	Down-regulation	β-Actin	qRT-PCR	Median	8	30	Tissue	OS
39	HULC	Wei Peng	2014	China	2006–2010	212/92	304	Up-regulation	GAPDH	qRT-PCR	No	7	60	Tissue	OS
40	MALAT1	Jiang-Hua Liu	2014	China	2010–2011	26/19	45	Up-regulation	GAPDH	qRT-PCR	Mean	8	40	Tissue	DSS
41	BC008363	Jiao Li	2014	China	2009- 2010	17/13	30	Down-regulation	GAPDH	qRT-PCR	Median	8	30	Tissue	OS
43	LOC285194	Yue-Chao Ding	2014	China	2004–2009	45/40	85	Down-regulation	GAPDH	qRT-PCR	Mean	6	60	Tissue	
50	C/EBPb	Chen-Song Huang	2017	China	2008–2013	54/29	83	Up-regulation	NO	qRT-PCR	Mean	7	100	Tissue	OS/DFS
50	LINC01133	Chen-Song Huang	2018	China	2015–2017	88/89	177	Up-regulation	NO	qRT-PCR	Mean	8	100	Tissue	OS/DFS
59	DUXAP8	Yifan Lian	2018	China	2007–2012	29/29	58	Up-regulation	GAPDH	qRT-PCR	median	7	60	Tissue	OS
67	CASC2	Yaqun Yu	2017	China	No	56/54	110	Down-regulated	RNU6B	qRT-PCR	Median	7	48	Tissue	OS
76	DANCR	Lei Chen	2018	China	No	86/120	206	Up-regulation	GAPDH	qRT-PCR	Mean	8	72	Tissue	OS/PFS
83	LINC00346	Wan-Xin Peng	2019	China	No	46/131	177	Up-regulation	GAPDH	qRT-PCR	No	4	80	Tissue	OS
103	XIST	Wei Wei	2017	China	No	32/32	64	Up-regulation	RNU6B	qRT-PCR	Median	8	30	Tissue	OS
109	ABHD11-AS1	X. QIAO	2018	China	2010–2013	72/75	147	Up-regulation	GAPDH	qRT-PCR	No	8	60	Tissue	OS
112	DLEU1	Song Gao	2018	China	2012–2017	39/23	62	Up-regulation	GAPDH	qRT-PCR	Median	8	60	Tissue	OS
114	FEZF1‐AS1	Zheng‐Lin Ou	2019	China	No	28/28	56	Up-regulation	RNU6B	qRT-PCR	Median	7	60	Tissue	OS
125	lnc-PCTST	Yandong Wang	2018	China	2015–2016	24/24	48	Down-regulated	β-Actin	qRT-PCR	Median	6	20	Tissue	OS
126	MACC1-AS1	Chen Qi	2019	China	No	49/49	98	Up-regulation	β-Actin	qRT-PCR	No	6	100	Tissue	OS
136	XLOC_000647	Hao Hu	2018	China	2015–2016	24/24	48	Down-regulated	β-Actin	qRT-PCR	Median	7	20	Tissue	OS
144	HOTTIP	Ozkan Balcin	2018	Turkey	2006–2014	61/39	100	Up-regulation	GAPDH	Q qRT-PCR	No	7	46	Tissue	OS
152	MSC-AS1	Yunpeng Sun	2019	China	No	23/22	45	Up-regulation	GAPDH	qRT-PCR	Median	6	36	Tissue	OS
159	Sox2ot	Zhonghu Li	2018	China	2012–2016	31/30	61	Up-regulation	NO	qRT-PCR	Median	8	50	Blood	OS
162	SPRY4-IT1	Yue Yaoa	2018	China	2011–2012	26/20	46	Up-regulation	GAPDH	qRT-PCR	No	7	60	Tissue	OS
169	GSTM3TV2	Guangbing Xiong	2019	China	No	100/80	180	Up-regulation	GAPDH & U7	qRT-PCR	No	8	80	Tissue	OS
172	SNHG15	X.-B. GUO	2018	China	No	82/89	171	Up-regulation	GAPDH	qRT-PCR	Median	8	60	Tissue	OS
185	HULC	Zheng-Lin Ou	2019	China	2012–2014	NO	60	Up-regulation	GAPDH	qRT-PCR	Median	8	40	Tissue	OS
186	lncRNA-UFC1	Peng Liu	2019	China	2012–2015	19/29	48	Up-regulation	U6	qRT-PCR	Mean	8	60	Serum	OS
187	FOXP4-AS1	Xiao-Guang Liu	2019	China	No	NO	112	Up-regulation	NO	NO	Median	4	50	Tissue	OS
188	PLACT1	Xiaofan Ren	2020	China	2008–2018	83/83	166	Up-regulation	NO	NO	Median	7	60	Tissue	OS
189	ENSG00000254041.1	Bo Chen	2020	China	2013–2014	35/35	70	Up-regulation	GAPDH	qRT-PCR	Median	4	50	Tissue	OS

*OS* overall survival, *RFS* recurrence-free survival, *DFS* disease free survival, DSS disease specific survival, *PFS* prognosis free survival, *TMED11P* trafficking protein 11, pseudogene, *lncRNA-ATB* long non-coding RNA-activated by transforming growth factor β, *AFAP1-AS1* actin filament-associated protein 1 antisense RNA 1, *UCA1* urothelial carcinoma associated 1 RNA, *CRNDE* Colorectal neoplasia differentially expressed, *HOTTIP* HOXA transcript at the distal tip, *Malat1* metastasis associated lung adenocarcinoma transcript 1, *CASC2* Cancer susceptibility candidate 2, *DANCR* Differentiation antagonizing non-protein coding RNA, *XIST* X-inactive specific transcript, *SNHG16* small nucleolar RNA host gene 16, *HULC* Highly up‐regulated in liver cancer, *qRT-PCR* quantitative real-time polymerase chain reaction, *NA* not applicable

### Association between lncRNAs expression and OS

**Fig. 2:**
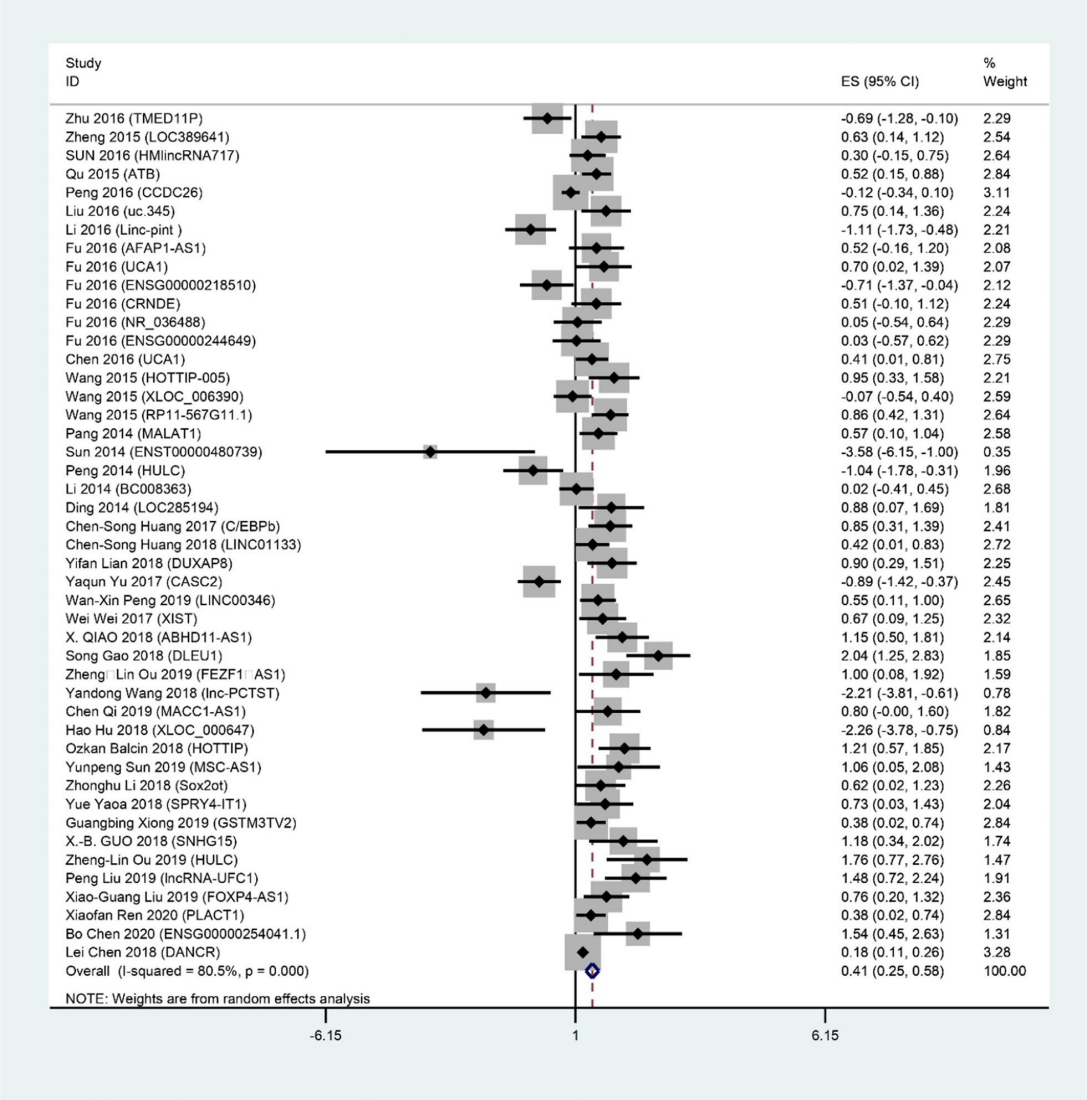
Forest plot for the association between lncRNAs expression and OS of pancreatic cancer patients

We conducted the present meta-analysis to figure out the value of aberrantly expressed lncRNAs in OS of 4691 PC patients from 39 studies. Statistical analyses represented significant association between the expression level of dysregulated lncRNAs and poor OS of PC patients in the relevant studies (HR = 0.41, 95% CI 0.25 ± 0.58, I^2^ = 80.5%, P = 0.000, random-effects) as well as this effect in these studies analyzed by univariate analysis (HR = 0.19, 95% CI − 0.156 ± 0.535, I^2^ = 0.0% P = 0.457) and multivariate analysis (HR = 0.262, 95% CI 0.207 ± 0.317, I^2^ = 81.7% P = 0.000) (Fig. 2), while a significant heterogeneity existed between studies (I^2^ = 80.5%, P = 0.000). Due to the presence of obvious heterogeneity, we performed subgroup analyses based on the ethnicity, molecular mechanisms and the expression level of lncRNAs in PC patients but similarly, heterogeneity was also assessed in our stratified analyses and there did not significant changes in heterogeneity after our subgrouping (Table 2).

**Table2:** Main results of subgroup analyses

Categories	Subgroups	n	HR	(95% CI)	Heterogeneity
All		46			80.5%
Ethnicity	China	45	0.25	(0.20, 0.31)	80.2%
	Another country	1	1.21	(0.57, 1.85)	–
Expression level	Up-regulation	35	0.32	(0.27, 0.38)	73.6%
	Down-regulation	11	− 0.54	(− 0.74, − 0.34)	71.9%
Molecular mechanisms	Metastasis	20	0.20	(0.14, 0.26)	80.7%
	Proliferation	31	0.23	(0.17, 0.29)	84.3%,
	Migration	5	0.73	(0.50, 0.96)	74.0%
	Invasion	20	0.21	(0.09, 0.33)	84.2%
	Tumorigenesis	10	0.08	(− 0.06, 0.22)	79.8%
	Apoptosis	11	0.27	(0.13, 0.41)	84.1%

### Association between lncRNAs expression and DFS

**Fig. 3:**
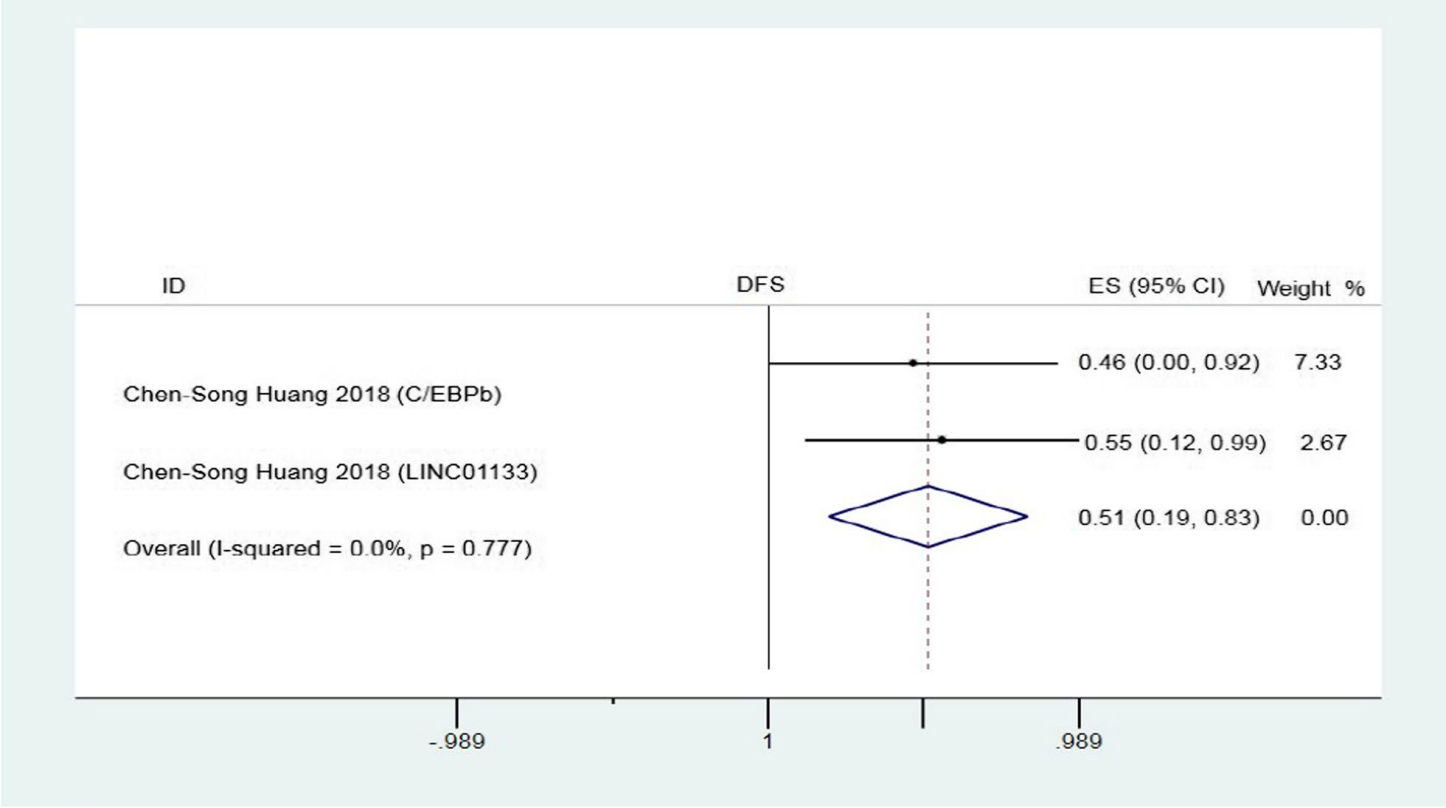
Forest plots for the association between lncRNAs expression and DFS of pancreatic cancer patients

The prognostic value of lncRNAs in DFS was explored in two studies including 260 patients. LncRNAs expression were significantly linked with DFS (HR = 0.51, 95% CI 0.19 ± 0.83, P = 0.00, fixed-effects; Fig. 3), while no significant heterogeneity was observed in these studies.

### Correlation of lncRNAs with clinicopathological characteristics of pancreatic cancer

Further stratified study grouped by clinicopathologic features exhibited that OS of patients with PC was markedly associated with gender (univariate analysis: HR = 0.04, 95% CI − 0.07 to 0.16, P = 0.344; multivariate analysis: HR = 0.01, 95% CI − 0.14 to 0.17, P = 0.868), Distance metastasis (univariate analysis: HR = 0.02, 95% CI − 0.53 to 0.57, P = 0; multivariate analysis: HR = 0.08, 95% CI 0.02 to 0.13, P = 0.0) Node metastasis (univariate analysis: HR = − 0.12, 95% CI − 0.34 to 0.11, P = 0; multivariate analysis: HR = 0.20, 95% CI 0.12 to 0.28, P = 0.0) and other clinicopathologic factors demonstrated in Table 3.

**Table 3:** Summary of the subgroup analyses of the association between OS and clinicopathological features in PC

Multivariate analysis					Univariate analysis					
	Included studies	HR (95% CI)	P value	I2 (%)	Effect model	Included studies	HR (95% CI)	P value	I2 (%)	Effect model
Gender	23	0.01 (− 0.14, 0.17)	0.868	0.0%	Fixed	10	0.04 (− 0.07, 0.16)	0.344	8.5%	Fixed
Distance metastasis	20	0.08 (0.02, 0.13)	0.000	74.7%	Random	1	0.02 (− 0.53, 0.57)	0.000	0%	–
Node metastasis	29	0.20 (0.12, 0.28)	0.000	65.9%	Random	5	− 0.12 (− 0.34, 0.11)	0.000	83.0%	Random
Differentiation	20	0.34 (0.22, 0.46)	0.145	25.4%	Fixed	12	0.15 (0.01, 0.29)	0.921	0%	Fixed
Neural and prineural invasion	13	0.24 (0.11, 0.36)	0.002	61.1%	Random	11	0.01 (− 0.01, 0.02)	0.178	28.0%	Fixed
Vascular invasion	9	0.36 (0.17, 0.56)	0.047	50.9%	Random	2	0.24 (− 0.14, 0.62)	0.339	0.0%	Fixed
TNM	30	0.07 (− 0.15, 0.29)	0.000	76.4%	Random	–	–	–	–	–
Stage	15	0.10 (− 0.00, 0.19)	0.000	69.8%	Random	4	0.48 (0.26, 0.70)	0.004	77.9%	Random

*HR* hazard ratio, *95% CI* confidence intervals

### Publication bias

**Fig. 4:**
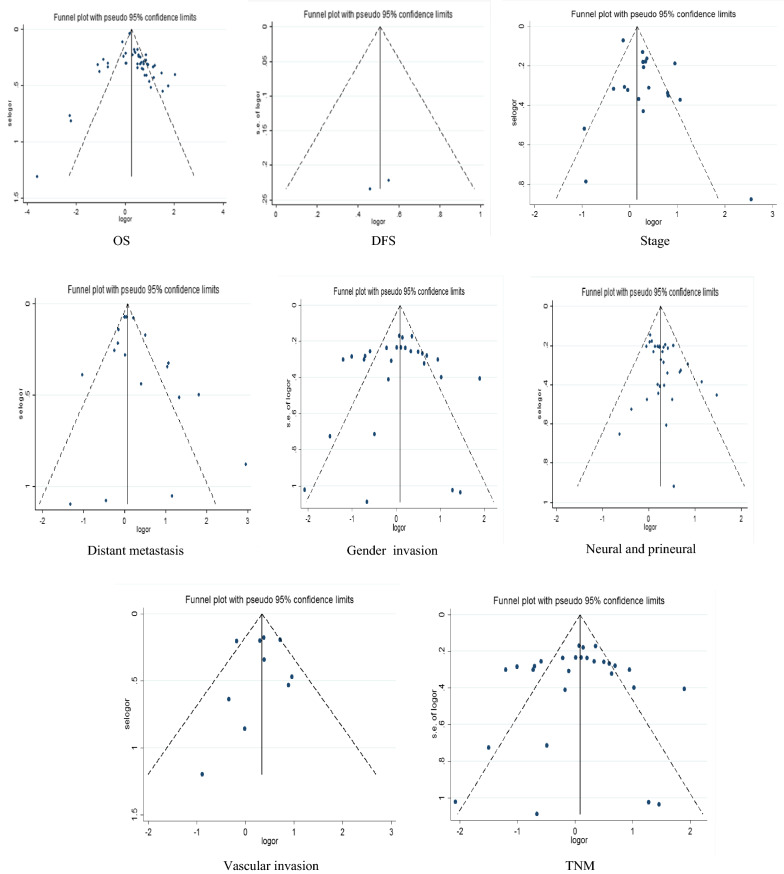
Funnel plot analysis for publication bias

The Funnel plot analysis was used to display asymmetry among the OS, DFS, distant metastasis, differentiation, gender, neural and prineural invasion, LNM, TNM and Stage (Fig. 4). Besides, no evidence of statistically significant publication bias observed by applying the Bgger tests and the Funnel plot analysis in combined prognostic studies.

### Sensitivity analysis

**Fig. 5:**
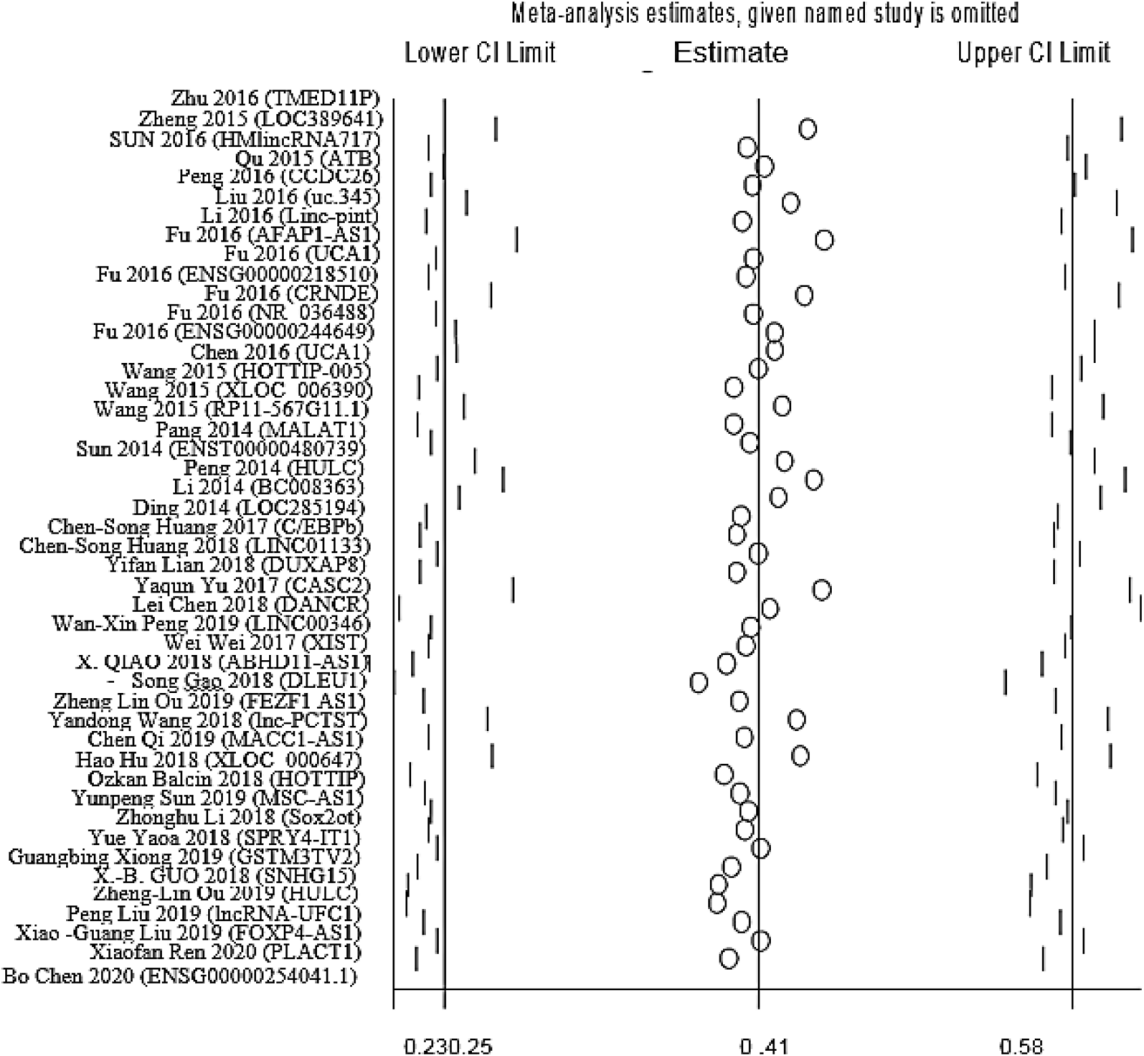
Sensitivity analysis of the effect of individual studies on the pooled HRs for lncRNAs expression and OS of PC patients

Sensitivity analysis was performed to discover the influence of the individual study on the pooled results by removing one single study from the overall pooled analysis. The results depicted that no individual study significantly changed the pooled HRs (Fig. 5) demonstrating that our analysis was relatively stable and reliable. Also sensitivity analysis showed that no individual study had great influence on final results of our meta-analysis.

## Discussion

Despite many advances in cancer research and treatment, the insidious onset of symptoms and extremely poor diagnosis of PC has still remained a controversial issue. The 5-year survival rate was estimated lower than 25% resulting in worse clinical outcomes in PC [63]. Imaging methods including, computed tomography, magnetic resonance imaging and endoscopic ultrasound are currently available methods used in the diagnosis and prognosis of PC. Moreover, a number of serum biomarkers, such as circulating tumor DNA and certain microRNAs are used in these regard [64, 65]. However, clinical application oh these methods in pancreatic cancer has been limited by their low specificity and sensitivity. Therefore, finding novel biomarkers are of most importance for early detection and more accurate treatment of this disease [66].

Over two past decades, numerous studies have focused on the potential roles of lncRNAs as contributors in various cell biological processes including gene and protein expression patterns. A growing body of evidence has verified the association between aberrant expressions of multiple lncRNAs with clinical outcomes for cancer patients. Notably, diagnostic significance of different lncRNAs profiling in digestive system tumors has been proved in numerous publications [67, 68]. So, in order to investigate the promising prognostic biomarkers for PC, as a high-degree malignancy of digestive system, the present systematic meta- analysis was performed to provide evidences to confirm potential association between altered lncRNAs and poor survival outcomes in PC. In this study, the information of 4736 PC patients was extracted from 39 studies conducted between 2014–2020. Our results represented altered lncRNAs is significantly linked with OS decline. Notably, we updated and augmented the reported results of meta-analysis carried out in 2017 with regard to the association between dysregulated lncRNAs and survival outcomes in PC [15].

In the current study, we assessed the prognostic role of different lncRNAs and their association with clinicopathological characteristics of PC. We found significant relation between altered expression of lncRNAs with poor OS period of PC (HR = 1.52, with 95% CI 1.04–2.22, and P = 0.031 in univariate analysis; HR = 1.55, with 95% CI 1.19–2.02, and P = 0.001 in multivariate analysis), suggesting that lncRNAs expression profile can be a prognostic biomarker of PC [14, 63, 69].Correspondingly, our stratified analysis evidenced that the clinicopathological factors as Gender, distance metastasis, node metastasis, differentiation, neural and prineural invasion, vascular invasion, TNM, Stage were remarkably contributed with OS of PC.

Moreover, large degree of heterogeneities among included studies were observed inspiring us to search its main causes from different aspects [70]. In this regard, we did subgroup analyses based on the ethnicity, molecular mechanisms and the expression level of lncRNAs in PC patients, however heterogeneity was also showed in our stratified analyses without any significant effect on heterogeneity.

Totally it could be concluded that lncRNAs expression profiling may serve as a helpful diagnostic and prognostic biomarker in of PC. So investigating the suitable single or panel of lncRNAs should be the focus of future studies [71–73].

However, it should be noted that there are several limitations in our meta-analysis including (1) The small sample sizes of the diagnostic meta-analysis as well as the limited clinical relevance of our results; (2) large heterogeneity in our analyses; (3) The HRs and 95% CIs from some of articles could not be directly obtained and were estimated by software, which may decline the overall accuracy of the pooled effects. Totally, results from our study did not fully show the real clinical significance of lncRNA signature in PC, and in order to obtain a decisive conclusion, further comprehensive meta-analyses are needed to confirm the strong association between the expression pattern of lncRNAs and outcome of PC patients.

## Conclusion

Altogether, our meta-analysis was updated and completed pervious reports to survey the prognostic value of lncRNAs and their association with clinical features of PC patients. Despite some above mentioned limitations, the present study revealed that lncRNAs could be used as potential prognostic markers for PC. However, more high quality and large-scale studies are still needed to validate the clinical utilities of lncRNAs in management of PC.

## Data Availability

Not applicable.
